# Improving fundamental movement skills in Hong Kong students through an assessment for learning intervention that emphasizes fun, mastery, and support: the A + FMS randomized controlled trial study protocol

**DOI:** 10.1186/s40064-016-2517-6

**Published:** 2016-06-16

**Authors:** Cecilia Chan, Amy Ha, Johan Y. Y. Ng

**Affiliations:** Department of Sports Science and Physical Education, Chinese University of Hong Kong, Shatin, Hong Kong

**Keywords:** Fundamental movement skills, Assessment for learning, Perceived competence, Enjoyment, Teacher support, Primary physical education, School-based intervention, Technology

## Abstract

**Background:**

Assessment for learning has been identified as an effective strategy to help children learn more effectively. Developing children to master basic movement skills in primary school requires formative assessments to inform instruction and learning. This study reports the rationale and methods for an assessment-based intervention that emphasizes fun, mastery and support (A + FMS) designed to improve fundamental movement skill (FMS) proficiency of primary schoolchildren.

**Methods/design:**

Utilizing a cluster randomized controlled trial, the A + FMS intervention was designed to improve FMS proficiency of Hong Kong Chinese schoolchildren. A target sample of 282 students or more from 10 Grade 3 classes (from five schools) will be recruited and randomly assigned into an experimental group or a wait-list control group. Competence motivation theory provided a framework for the intervention that emphasizes fun activities to develop basic fundamentals, improving mastery of movement, and providing support for teaching and learning skills. Primary outcome measures are the raw scores of six objectively measured FMS (i.e., jump, hop, skip, dribble, catch, and overhand throw). Secondary outcomes include self-reported measures: enjoyment in physical education, perceived physical competence, perceived skill competence, and perceived social support. Teachers in the experimental group are required to attend a six-h training workshop and integrate 550 min of assessment for learning strategies into their physical education lessons. Resources such as videos, skills checklists, and equipment will also be provided to support children to accumulate extra learning and practice time after school. The rate of changes in primary and secondary outcomes across the experimental and control groups will be compared to determine the effectiveness of the program.

**Discussion:**

The A + FMS is an innovative school-based intervention targeting improvements in movement mastery by supporting physical education teachers in FMS instruction and assessment practices. The findings from the study may be used to guide pre-service teacher education and continuous professional development in FMS teaching and assessment.

*Trial registration* CUHK_CCRB00479

## Background

Fundamental movement skills (FMS) competency, including locomotor and object control skills, has been identified as a key mediator for the changes in children’s physical activity (PA) and cardiorespiratory fitness (Cohen et al. 2015). An increasing amount of evidence suggests that the development of motor skill competence is an important underlying mechanism that promotes engagement in PA (Castelli and Valley 2007; Barnett et al. 2011; Stodden et al. 2008). According to competence motivation theory (CMT) (Harter 1978), children who perceive themselves to be competent in PA and influenced by significant adults and peers would have the interest and desire to engage in various types of activities or pursue various challenges. In order to increase children’s competence and confidence in FMS, movement skill programs that involve quality instruction and feedback, adequate skill practice opportunity, and fulfilling and fun activities from qualified personnel have been identified as a promising approach (Morgan et al. 2013).

Around the age of transition to the upper primary years (i.e., Grades 4–6, or 8–10 years old), children should be able to achieve a mature pattern of movement in fundamental skills (Gallahue and Cleland-Donnelly 2007). Mastery of FMS components in this age group is crucial if children are to graduate with a level of competence that enables them to live a physically active and healthy lifestyle in early secondary school years (Hardy et al. 2013). Research has shown Hong Kong children’s active behaviors are extremely limited in both inside and outside of school due mainly to environmental reasons (Johns and Ha 1999). Physical education (PE) plays an important role in the promotion of FMS proficiency in children, and PE teachers become the most significant change agents to provide instructional support and skill-learning opportunities during class time. Social support from teachers has been a targeted strategy in school-based PA and fitness interventions (Eather et al. 2013), and it may also provide motivational reinforcement and encouragement in the acquisition of and improvements in FMS. Emerging research suggests that providing mastery-oriented PE environments that emphasize on learning and mastery of skills could support the learning of movement skills (Martin et al. 2009; Valentini and Rudisill 2004). However, researchers have found that PE teachers have limited content knowledge of how to develop FMS and the ability to improve the motor performance of their students (Ennis 2011; Lounsbery and Coker 2008).

The provision of a good PE program in primary years is critical in ensuring that students in this stage develop and demonstrate proficiency in FMS. PE should be about teaching new skills and be more than just providing them with fun PA. PE teachers are required to possess the knowledge and skills necessary to demonstrate competent movement performance and implement progressive and sequential instruction. According to the National Association for Sport & Physical Education’s National Standard for Physical Education (Society of Health and Physical Educator 2010), assessment of student learning plays an important role in motor skill instruction. This assessment requires teachers to possess assessment-related skills and knowledge to design movement content of the lessons in accordance with existing abilities and to provide appropriate feedback for all learners with formative assessments.

In the past two decades, the call for a change in assessment practices in education worldwide to improve teaching and learning (Berry 2011) has contributed to the need for assessment reform in Hong Kong (Hong Kong Education Commission 2000). To promote better learning, schools are encouraged to put more emphasis on Assessment for Learning (AfL, also known as formative assessment) as an integral part of the learning, teaching, and assessment cycle. Assessment has more to do with helping students grow than with cataloging their mistakes. The main strategies considered important for AfL include sharing learning goals, effective questioning, formative feedback, peer and self-assessment, and using assessment information to improve future student performance (Flórez and Sammons 2013; Black et al. 2003; Black and Wiliam 2006). These strategies are conducted during daily classroom practice to allow teachers to meet diverse student needs, and enable feedback to improve learning and inform instruction (Black et al. 2003).

Unlike teachers of academic subjects using written or oral tests, PE teachers focus primarily on movement, physical activities, and sports skills, assessment in PE cannot take place without regular observation of movement. However, observation, by itself, is not assessment. Tomlinson suggests that “assessment is today’s means of understanding how to modify tomorrow’s instruction (Tomlinson 2014).” In other words, assessment and instruction are inseparable. Therefore, AfL that emphasizes the value of feedback to modify instruction and concerns with the progress of learners has the potential to improve motor skill proficiency among schoolchildren.

Motor skill development do not simply develop as a result of age. Children must be provided with quality instructions and feedback, and sufficient opportunities for practice to develop FMS proficiency (Hands 2012). This require teachers to be equipped with effective instructional and assessment approaches to bring about change in children’s motor skill development. Given that assessment is a weak component in PE (Wood 2003) and most teachers reported a lack of skills or knowledge for incorporating assessment into their programs (Morgan and Hansen 2007), enhancing PE teachers’ assessment literacy and practice in their daily teaching is needed to promote student learning. Researchers have found that improvements are substantial when teachers are supported to teach FMS (van Beurden et al. 2003; Mitchell et al. 2013). These findings provide evidence that school-based FMS teacher support intervention may be an effective way to improve FMS competencies and mastery. However, many FMS school-based interventions focus on supporting teachers by providing mentorship (van Beurden et al. 2003) or by modifying their instruction skills and learning environment (Miller et al. 2015), rather than by enhancing in-service teachers’ assessment competence to promote student learning.

The long-term aim of promoting lifetime PA requires a long-term approach to provide adequate training, facilities, and equipment for physical educators to be confident in teaching FMS. Assessment must be viewed by physical educators as necessary to increase the accountability of PE (Wood 2003). That is, if sound assessment practices are not in place in teaching and learning FMS, its value and importance in PE programs might be eventually diminished. To date, no research to our knowledge has attempted to improve children’s FMS competency by enhancing in-service teachers’ assessment competence (Riethmuller et al. 2009; Morgan et al. 2013; Lai et al. 2014). School-based FMS interventions designed to enhance PE teachers’ assessment literacy and practice in their daily teaching are warranted. Although evidence on school-based interventions in Hong Kong is increasing, it is limited and focused mainly on improving FMS proficiency in children with developmental coordination disorder (Capio et al. 2015) or on increasing activity solely during PE classes (Ha et al. 2015). As many children entering adolescence have not yet mastered the basic movement skills (Hardy et al. 2013), helping children practice FMS at home, other than the school environment, is also important. A take-home worksheet or specific homework activities can be assigned to students, or including some parental involvement to help practice the skills being taught during PE can provide additional opportunities to improve FMS (Gallahue and Cleland-Donnelly 2007). Therefore, this study focuses on increasing children’s FMS proficiency through teacher support in instruction and assessment of FMS, and encouragement of FMS practice in and outside of PE.

The main objective of the current trial is to examine whether the implementation of AfL strategies in PE classrooms can improve FMS proficiency in jumping, hopping, skipping, catching, dribbling, and overhand throwing. Changes in children’s enjoyment of PE, and perceptions of physical competence, movement skill competence and teacher support as secondary outcomes will also be used to evaluate the effectiveness of the intervention. This school-based teacher support intervention will be led by the PE teachers after receiving a six-h training workshop relating the teaching and assessment of FMS proficiency. Specifically, teachers assigned to the experimental groups will have to embed AfL strategies into FMS teaching and learning for 550 min of PE class time. A systematic review and meta-analysis of the benefits of FMS interventions among the youth found that interventions on average offer between 8 and 195 h of instruction and run for 12 weeks (median) (Morgan et al. 2013). Given the low time allocation (i.e., 5.41–5.9 % of total lesson time) and different time allotment for PE among Hong Kong primary schools (i.e., 5-day week or 6-day cycle with single or double periods per week/cycle) (Wang and Kirkpatrick 2015), 550 min of assessment-driven instructions (i.e., integrating assessment into instruction) is considered appropriate. Conversely, teachers of classes allocated in the wait-list control will employ normal teaching and assessment practices during the intervention period. Compared with students receiving usual practice, we therefore hypothesize that students in PE lessons in which teachers integrate AfL strategies into FMS teaching and learning for 550 min will demonstrate greater increases in FMS, perceived physical competence and movement skill competence, enjoyment in PE, and perceptions of teacher support.

## Methods/design

### Trial design

The designed school-based intervention (September 2015 to February 2016) will be evaluated using a cluster randomized controlled trial. Clusters (i.e., classes) will be randomized to receive either the “A + FMS” intervention or carry on with the usual practice, with a 1:1 allocation ratio. This study will be conducted in compliance with the principles of Declaration of Helsinki. Ethics approval for the study was obtained from the Survey and Behavioral Research Ethics of The Chinese University of Hong Kong, and it is registered with the CCRB Clinical Trials Registry, CUHK, under number CUHK_CCRB00479. Following the initial recruitment processes, baseline assessments will be conducted at participating schools. Principals and PE teachers will provide written informed consent. All participants are required to return a signed informed consent letter from their parents prior to their participation in this trial.

### Sample size calculation

The primary outcome variable in this study is FMS. Before recruitment, a power calculation was conducted to determine the sample size necessary to detect meaningful changes in the total raw scores of six FMS (i.e., jumping, hopping, skipping, catching, dribbling, and overhand throwing). Using an alpha of 0.05 and power of 80 %, the calculations were based on the effect sizes reported in a recent systematic review and meta-analysis (Morgan et al. 2013). The expected effect size of the intervention on overall FMS skill proficiency was found at 1.42 [standardized mean difference (SMD)]. With reference to these results, a prudent estimation of effect size (SMD = 1.20) was used for calculation, as the treatment of this study is comparatively short term (i.e., 550 min). The required sample was calculated to be 24. To account for the clustering effect, this number was then multiplied by a factor of 1 + (*m*−1)*ICC, where *m* is the average cluster size and ICC is the intraclass correlation coefficient. Based on previous work conducted by the authors, the ICC was estimated at 0.37. With an estimated average class size of 30, at least 282 participants will have to be recruited. Therefore, students from ten classes will be invited to take part in the trial.

### Recruitment and study participants

**Fig. 1:**
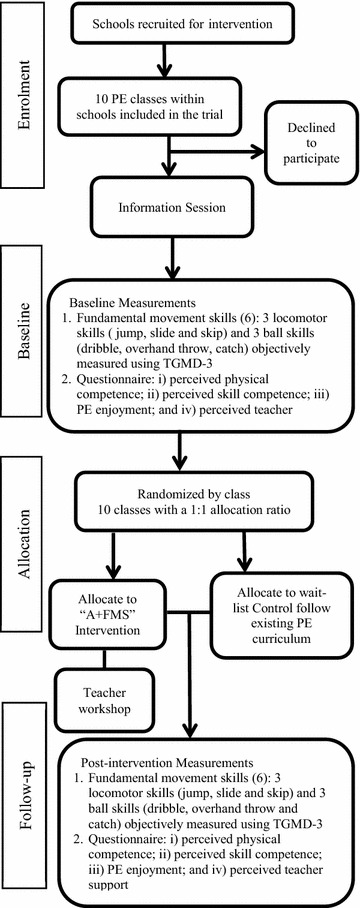
Flow diagram of the A + FMS study protocol

Primary schools will be invited to participate in this study. Our recruitment goal is 10 Grade 3 classes, with a total of 282 students or more. A briefing session will be convened to introduce the program to the participating PE teachers. Each participating school will be invited to provide two to three classes of Grade 3 students (typically 9 years old). All students from Grade 3 will be eligible to participate in the program if they do not currently have a pre-existing injury or medical condition that prevents testing or training. Figure 1 depicts the flow of “A + FMS” study protocol. All eligible participants will complete baseline and immediate post-intervention assessments to determine intervention effects.

### Blinding and randomization

The allocation of treatment will be blinded from student participants, and research assistants who will conduct FMS testing sessions and administer the questionnaire. Teachers will not be blinded to group assignment, as they will attend the FMS training workshop and be required to implement the intervention. Randomization by cluster (i.e., class) will be performed at the completion of the baseline assessments, and the ten participating classes will be randomly assigned to the A + FMS intervention (five classes) or a wait-list control group five classes). After the baseline measures are taken, a grouping tool, “Grouping Wizard,” developed by Education Bureau, HKSAR Government, will be used to randomly allocate classes into one of the two treatment conditions. Teachers will be informed about the allocation results but not about the group allocation mechanism.

### Intervention

The “A + FMS” program is grounded on Harter’s CMT, and its implementation will be guided by the AfL principles and strategies (Black et al. 2003; Assessment Reform Group 2002). By targeting the constructs of CMT, this school-based intervention is designed to promote fun, mastery, and support for improving FMS proficiency. Harter’s CMT provides a framework for the intervention that emphasizes student-centered learning, fun activities to develop basic fundamentals, improve mastery of movement, and provide support for teaching and learning. Therefore, we aim to provide children with knowledge and skills required to produce mastery, and positive feedback given for improvement to nurture perceptions of competence and control, positive affect and intrinsic motivation.

A six-h training workshop will be provided to the teachers. The objectives of the workshop are for teachers to (1) refresh their knowledge and skills in the teaching and assessment of FMS; (2) become familiar with the assessment criteria of the six FMS (i) locomotor skills: jump, hop, and skip and (ii) ball skills: dribble, catch, and overhand throw; (3) share teaching techniques and practical ideas to improve the development of children’s FMS; (4) develop an understanding of the AfL concept; and (5) plan for the use of AfL strategies into the learning and teaching of FMS within the PE context. The teacher workshop will be led by the lead author, who is a dance instructor with an accredited teaching qualification and highly experienced in teaching dance and rhythmic movement for children of all age levels. Therefore, the lead author understands the development of a child’s fundamental movement skills and how to support mastery of movement using fun and rhythmic activities. Teaching and learning activities include lecture, practical session, demonstration, peer evaluation using videos, questions and answers, and group discussion.

During the workshop, teachers will be instructed about the testing protocol and the performance criteria of each of the six selected FMS (i.e., jumping, hopping, skipping, catching, dribbling, and overhand throwing) proposed in The Test of Gross Motor Development, 3rd edition (TGMD-3) (Ulrich 2016). The teachers will also receive training in FMS analysis for feedback, instruction, and assessment. TGMD assesses the qualitative aspects of motor skills. The major advantage of qualitative assessment is to inform teachers or movement professionals on which specific components of a skill an individual needs to practice, thus making the assessment undertaken more meaningful than quantitative methods (Hands 2012). Practical sessions, peer evaluation, and group discussions will be led by the lead author to examine how to teach and assess the selected FMS using the performance criteria. Teachers will also be guided through and familiarized with the AfL strategies for implementing them in their PE lessons. Instructional information, worksheets, and video links containing information about the key components of the particular FMS will be provided to facilitate effective questioning and self- and peer evaluations throughout the intervention.

Once trained, teachers in the experimental group will be asked to embed AfL strategies introduced in the Teacher Manual into FMS teaching and learning for a total of 550 min of PE class time. As the PE lessons of the participating schools range from 45 to 70 min per week, the program will last for at least 8 weeks up to a maximum of 12 weeks. Students will be encouraged to complete FMS progressive activities and assessments through the help and support of their parents and/or caregivers. Audio visual aids, such as videos and an illustrated practice handbook with colorful images and information, about the key elements of the particular FMS being stressed during PE will be provided to encourage further self-practice outside of the school context with peers, friends, siblings, and family. Two different sizes of spongy balls for throwing and catching are also provided to practice the skills described in the program. PE teachers in the control group will follow their existing school-based PE curriculum without additional resources and support in FMS instruction.

### Components of A + FMS

Assessment, fun, mastery, and support are the four major components of the A + FMS program. The key features of this program include emphasis on the ongoing, student-centered assessments to improve instruction and learning; use of technology to make the teaching–learning process more meaningful and fun; quality instruction and practice coupled with constructive feedback to improve mastery of movement; and provision of resources to support teachers’ ability to teach and enable students to learn. The focus of this intervention is on promoting children’s FMS proficiency, enjoyment in PE, perceptions of physical competence, skill competence, and teacher support. The program overview is summarized in Table 1. Details of the intervention components (i.e., assessment, fun, mastery and support) that may produce these desired outcomes are highlighted as follows:

**Table 1:** A + FMS intervention components, strategies, contents and alignment with CMT constructs

Components	Key features of the strategy	Program contents	CMT constructs
Assessment	Formative student-centered assessment to integrate AfL strategies into instruction	Introduce the assessment criteria to learners by demonstrating the standards required Check students’ understanding through the effective use of questioning Provide the necessary guidance and support to learners on an individual basis and provide oral feedback Provide self-assessment opportunities to identify the gap in their own learning to aid learning, promote progress, and contribute to the self-management of learning Provide peer-assessment opportunities to apply the assessment criteria to work produced by their peers; peer assessment using the predefined assessment criteria is the next stage to evaluate learner understanding and consolidating learning	Perceived control over learning Mastery and competence Perceived competence Social support for learning to learn Engagement and motivation
Fun	Pedagogy—technology integration using fun activities to develop basic fundamentals	Present instructional videos as part of the instructional process to provide a learning environment that is colorful, engaging, and interactive. With the QR Code links, teachers will be able to show their class, or on a tablet or iPad, what the skill looks like in action Introduce simple, enjoyable and convenient FMS activities for PE lessons to make movement skill learning easier and more fun to ensure that the fundamentals of the skill are acquired Provide sample videos of simple and fun activities for home practice to strengthen student learning anywhere, anytime Praise students’ efforts and emphasize mastery rather than winning or losing. Learning and practice are about having fun Encourage home practice with peers, friends, siblings, and family after school with the use of videos and an illustrated practice handbook to enable students’ self-regulated learning and to make improvements independently	Perceived control over learning Mastery and competence Perceived competence Social support for learning to learn Affective reactions (perceived enjoyment and satisfaction) Engagement and motivation
Mastery	Quality instruction, practice and feedback to improve mastery of movement	Clear and specific success criteria on manageable chunks of learning to enable students to progressively achieve a level of mastery, be successful and develop self-competence Require students to have their work regularly checked against a list of criteria on their own and by another student to reflect and make progress in movement mastery. Peer and self-assessment opportunities will demonstrate what is being learned and identify areas for improvement Provide varied practice activities and encourage sufficient practice in and out of school to ensure mastery Effective questioning and feedback from teachers to ensure students’ skill development is continually being refined	Perceived control over learning Mastery and competence Perceived competence Social support for learning to learn Affective reactions (perceived enjoyment and satisfaction) Engagement and motivation
Support	Provision of resources and equipment to support teaching	A teacher manual designed to support teachers in teaching and assessing FMS and in integrating AfL strategies into their PE lessons. Instructional information and pictures of the observable components of each skill, checklists, and assessment forms will be provided The selected FMS are presented in short videos. Each video demonstrates the performance criteria of each FMS, including the slow motion of key performance indicators necessary for improving children’s movement skill proficiency An illustrated practice and assessment handbook is designed to motivate and guide students to gradually increase the level of difficulty and improve the individual skills with parental support Students are provided with equipment (small spongy balls) to practice the skills described in the program in and out of school Teachers receive regular support from the researcher through a messaging application for sharing and answering questions A follow-up meeting with teachers during the middle of the program to monitor students’ progress and attainment of goals, as well as to provide support and feedback to teachers	Perceived control over learning Mastery and competence Perceived competence Social support Affective reactions (perceived enjoyment and satisfaction) Engagement and motivation

*FMS* fundamental movement skills; *AfL* assessment for learning

Assessment is intertwined with the teaching process and is not separate from teaching. It provides feedback to adjust ongoing teaching and learning to improve students’ achievement (Black et al. 2003). Unlike summative assessment, AfL takes formative assessment (which is ongoing) during day-to-day classroom practice to provide teachers with information on which to base future learning episodes. The information provided will inform students directly about their adequacy in learning and performance, and will also provide teachers and students with improvement direction. It brings students into the assessment process as major decision makers and contributors to their own learning. As students begin to self-assess using formative assessment information, they assume greater ownership and control of their learning. AfL places considerable emphasis on the importance of teachers sharing learning objectives or assessment criteria with students. Students are informed about the criteria and expectations at the beginning of a unit. They understand what they are trying to learn, why, and what is expected of them. Both teachers and students are focused on the criteria that the work will be assessed against. As a level of competence is identified, students are better able to assess their progress toward a set of criteria to identify and experience success, and to remain motivated to carry on learning the tasks (Lund and Veal 2013). To help students to make plans for further improvement, teachers are required to use effective questioning techniques, observations, and timely and quality feedback on the learning objectives (or assessment criteria). The clear and explicit standards of performance also enable students to make judgment about others’ work. Clarke (2008) suggests that sharing and agreeing on the success criteria can help to cultivate independent learners, provide effective feedback, and create confident pupils both in the classroom and in life beyond the classroom (Clarke 2008). Therefore, such an approach to assessment is important for PE teachers to guide their instructional decisions and for students to foster competence and independence in learning FMS.

Enjoyment has been linked to perceived competence and mastery (Wallhead and Buckworth 2004) and to PA participation levels for children and adolescents (Sallis et al. 2000). Studies have shown that lack of enjoyment and perceived physical competence are the reasons children choose not to be active (Carroll and Loumidis 2001; Salmon et al. 2003). Children find PA fun and challenging when they can experience success. Programs that help primary schoolchildren feel more physically competent and confident to be active and that generate fun and autonomy are more likely to optimize children’s motivation to engage in the intervention (Jago et al. 2014). Nowadays, technology is present in the everyday lives of young children. Children see technological tools as fun, and they become more motivated in learning PE and achieving the techniques with audio visual aids (Grout 2009). A skill that is correctly executed and viewed repeatedly is considered to give positive feedback for correction and perfection of motor skills. The inclusion of technology in PE has the potential to promote effective teaching and learning. NASPE clearly supports the potential of technology as an effective tool for enriching PE instruction (National Association of Sport and Physical Education 2009).

The level of mastery of FMS represents the foundational behavioral competencies for PA participation (Gallahue et al. 2012). FMS are most successfully acquired during the elementary school years, and mature forms of these skills are the basis for all sports skills. Teachers should aim to have students master a skill rather than just experience it. Children who do not master these basic skills are less able and often less willing to participate in PA during adolescence (Barnett et al. 2009). The teaching of FMS requires teachers to identify the skill components and provide specific feedback to students. The identification of common errors associated with learning specific skills enable teachers to provide students with appropriate feedback to refine and improve their mastery level. Sufficient practice opportunity and successful learning experience may also be provided for children to master a skill and to continue attempting more challenging tasks. Attempts at mastery engagement are essential for building children’s perception of their competence. If they have successful attempts, they will enjoy the tasks and feel competent and highly motivated (Harter 1978). Lack of confidence in the physical domain will cause children to avoid activities that expose them to “public failure.” Therefore, the A + FMS program aims to improve children’s actual and perceived competence and to contribute in the fight against the dramatic decline of PA levels during adolescence.

The provision of equipment and the distribution of printed and audiovisual educational materials for teachers and students are some of the components highlighted in the systematic review of school-based PA interventions by Dobbin and colleagues (Dobbins et al. 2009). They were delivered effectively to increase PA among elementary schoolchildren. For effective FMS teaching, students should have the opportunity to practice their movement skills using a variety of equipment, such as bean bags and different-sized balls for catching and throwing. The equipment used varies the complexity of the skill (Griggs 2012). Furthermore, competency in observational skills and motor skill analysis is important for developing pedagogical competency in PE. However, primary PE teachers lack the confidence in teaching skills, and the availability of training opportunities still affect the quality of provision (Sloan 2010). The limited resources available in primary schools, coupled with the lack of expertise to develop and execute lessons, continue to be an ongoing concern. Interventions that provide professional learning opportunities, particularly teaching FMS, are urgently needed for sustainable practice (Lubans et al. 2012).

### Outcome measures

The primary outcome of the study is students’ movement skill proficiency in six FMS, including three locomotor skills (jump, hop, and skip) and three ball skills (catch, dribble, overhand throw). TGMD-3, a revised version of TGMD-2 and is due for release, will be used after confirmation with the developer (Personal Communication, Professor Dale Ulrich 17–19th January 2015). TGMD-2 is a well-validated standardized test commonly used to assess FMS of children aged between 3 and 10 years (Ulrich 2000), and it covers the period when the most dramatic changes in a child’s gross movement skill development occur (Cools et al. 2009). TGMD-2 has been reported to be a reliable and valid assessment for Hong Kong children (Pang and Fong 2009; Wong and Yin Cheung 2010).

Preceding the assessment of each skill, the research assistant will present an accurate movement demonstration video clip on a smartphone or tablet to the group with a brief verbal description of the skill. The use of visual demonstration is to ensure the accurate demonstration of the skill and to minimize any discriminatory practices among the testers. A two-h training workshop will be provided for the research assistants to gain competence in the assessment procedures prior to research. They will rate the videos of children performing the six skills, and each skill comprises three to five performance criteria which qualitatively describe a mature movement pattern of the skill performance. Participants will be given two trials for each FMS. If the behavioral component will be presented, one mark will be given; otherwise, no mark will be given. The scores of the two trials will be totaled to obtain a raw score for each skill. The sum of scores from the six skill tests will be used as the primary outcome of the trial.

Secondary outcomes include students’ perceived physical competence, perceived movement skill competence, enjoyment in PE, and perceived teacher support.

#### Perceived physical competence

The Athletic Competence subscale of the Self-Perception Profile for Children (Harter 1985b) (SPPC–6 items) will be used to assess the participants’ subjective evaluation of their athletic ability. For example, “Some kids wish they could be a lot better at sports,” but “Other kids feel they are good enough at sports.” First, the student will decide which of the two statements best described him/her, and then choose if the statement is “sort of true” or “really true” for him/her. This structure design decreases the tendency to give socially desirable responses and provides participants with a range of response choices (Harter 1985b). The SPPC was found to be a reliable and valid self-report measure for assessing children’s self-perception, and the observed coefficient (alpha) of the athletic competence was .80 (Harter 1985b).

#### Perceived movement skill competence

The pictorial scale of perceived movement skill competence for young children (Barnett et al. 2015) will be used to assess students’ skill perceptions of the six FMS (jump, hop, skip, dribble, catch, and overhand throw) objectively measured using TGMD-3. This instrument was modified from the format and item structure of Harter and Pike’s instrument (Harter and Pike 1984). For example, “The boy isn’t very good at skipping” was changed to “This boy is pretty good at skipping.” With separate cartoon illustrations provided for boys or girls performing the skill competently and less competently to be presented for each skill, this pictorial scale has acceptable face validity, good test–retest reliability (object control ICC = 0.78, locomotor ICC = 0.82, and all 12 skills ICC = 0.83), and internal consistency (alpha range = 0.60–0.81) in an Australian sample (Barnett et al. 2015). A score of four reflects the highest perceptions of competence and a score of one reflects the lowest perceptions of competence.

#### Enjoyment in PE

PE enjoyment will be measured using the PE Enjoyment Rating Scale (Prochaska et al. 2003). This face scale provides an indication of the direction and intensity of PE enjoyment. The response options are six “sad/happy” faces, from a frowning face (coded 1) to a smiling face (coded 6), for the question “How do you feel about PE classes?”

#### Perceived teacher support

Students’ perceived teacher support will be measured using Harter’s Social Support Scale for Children (Harter 1985a). The subscale includes six questions to assess the degree to which teachers help them if they are upset, help them do their very best, care about them, are fair to them, and treat them as a person. Similar to Harter’s SPPC (Harter 1985b), children are asked to read two statements and decide which one is more like them. For example, “Some kids don’t have a teacher who helps them to do their very best BUT other kids do have a teacher who helps them to do their very best.” Then, students decide if the statement is sort of true or really true for them. The scores are coded as follows: Really True for Me = 1, Sort of True for Me = 2, Sort of True for Me = 3, and Really True for Me = 4. The higher the score is, the greater the child’s sense of teacher support. This self-report subscale is appropriate for elementary schoolchildren aged 8–13 (grades 3–6), and the internal consistency reliability is 0.82 (Harter 1985a).

All the above measurements will take place at the children’s school during the scheduled PE lessons. Participants will be guided through each question in each section of the questionnaire by the trained research assistants to provide clear instructions and to clarify if the participants do not understand the questions.

### Process evaluation

A number of process measures will be used to ensure that the intervention is delivered and implemented as planned. A range of process data will be collected to complement the outcome data. (i) Pre-workshop/post-workshop evaluation forms will be used to measure the initial knowledge level of the teacher before the workshop, what they learned through the process of completing the workshop, and their satisfaction with their learning experience. (ii) Any of the two lessons of each participating teacher will be video-taped and evaluated against an AfL strategies checklist (i.e., sharing learning objectives, effective questioning, formative feedback, and self- and peer evaluation). (iii) The lead author will provide suggestions and feedback only to the experimental group teachers for each observation, and these teachers are required to complete the AfL strategies checklist and the lesson content record sheets. (iv) Teachers in the experimental group are encouraged to assess any five of the students’ FMS development and performance while teaching a particular skill during the lessons using the performance criteria assessment sheets for teachers. (v) A one-h mid-program review meeting will be scheduled with the experimental group teachers to share feedback about the intervention and to provide further support. (vi) Students’ practice and assessment handbook will be collected at the end of the intervention to determine students’ involvement in the completion of after-school FMS practice tasks and assessment activities. (vii) Teacher satisfaction with all intervention components will be determined using a post-intervention questionnaire at the completion of the study.

### Data analysis

To account for clustered nature of the collected data, multilevel modeling methods will be used for data analysis. 3-level (time within student within class) will be evaluated for both primary and secondary outcomes. Specifically, a 3-level regression with random intercepts and random slope for the time (baseline = 0; follow up = 1) variable will be examined, with independent variables of time, group (experimental = 1; control = 0), and time*group. A significant time*group term will denote the presence of intervention effect. Multiple analyses will be conducted by centering the Time variable at baseline and follow up to detect group differences in measured outcomes at the two time points, respectively.

## Discussion

Increasing evidence shows that school-based FMS interventions delivered by PE teachers and that provide professional learning opportunities for teachers are effective in improving FMS proficiency (Mitchell et al. 2013; Cohen et al. 2015; Morgan et al. 2013). The A + FMS is an innovative school-based intervention targeting improvements in movement mastery by supporting PE teachers in FMS instruction and assessment practices. This is the first randomized control trial to specifically target FMS proficiency as the primary outcome in primary school PE settings through substantial support for teachers’ assessment competence (Lai et al. 2014). Furthermore, the theoretical framework based on competence motivation theory provides detailed pedagogical approaches to facilitate effective integration of AfL strategies in school PE.

AfL comprised several empirically supported components has been recognized as central to classroom practice (Black et al. 2003), but to the authors’ knowledge, its utility and efficacy in the field of PE have not yet been tested through interventions using rigorous methodologies. It is a novel component of A + FMS intervention to focus on AfL strategies to assist children in the development of basic movements and self-perceptions of physical competence, movement skill competence, teacher support, and enjoyment in PE. Furthermore, by giving children printed handouts, audio-visual materials and equipment, the A + FMS aims to encourage and enable children to learn and practice FMS inside and outside of the classroom. The findings of this study will inform current literature regarding the effectiveness of the provision of substantial support for PE teachers in offering effective and joyful FMS learning experiences for primary school students.

Given that the primary school years are considered the optimal time to develop FMS and the current issues on FMS teaching in primary schools, the details described in this paper, such as the rationale, timeline, study design, and protocol overview of the A + FMS intervention, may be beneficial to PE educators or other researchers who are looking for novel strategies to help children overcome deficiencies in FMS proficiency. By using a combined approach, this trial involves teachers and with the assistance of the researcher, as well as indirectly involving parents to complement and support children the translation of knowledge and skills from school PE to the home environment. This approach not only offers a forum of support for teachers who may not be confident in delivering the intervention, but also ensure sustainable practices in the school context (Riethmuller et al. 2009). More importantly, the presented framework that addresses teachers’ needs for professional learning opportunities, and the strategies formulated to promote FMS development within existing PE lessons and outside school hours is replicable and relatively easy to scale.

This paper has reported the rationale and study protocol for an assessment-based intervention that emphasizes fun, mastery, and support for improvement of FMS proficiency among primary schoolchildren in Hong Kong. As incorporating a number of novel strategies to improving FMS in schoolchildren, the findings from the study may have important implications for teaching and learning of these basic skills. In view of the lack of FMS proficiency among children (Hardy et al. 2013) and a paucity of FMS interventions (Riethmuller et al. 2009), further understanding on professional learning of primary PE teachers to develop student FMS is necessary. A + FMS intervention that has a strong theoretical foundation will provide compelling evidence of using ongoing assessments and teacher support to improve instruction and to help the student mastering of movement skills. When movement skill assessment becomes part of a quality PE program, teaching and learning strategies will guide all students to acquire the knowledge and skills necessary to develop and improve their skill competency. Furthermore, the findings will ascertain whether the A + FMS strategy is a promising approach to enable children to improve FMS proficiency and to be delivered on a larger scale in primary schools.

## Abbreviations

A + FMS: assessment-based intervention emphasizes fun, mastery and support
AfL: assessment for learning
CMT: competence motivation theory
FMS: fundamental movement skills
PA: physical activity
PE: physical education
SPPC: self-perception profile for children
TGMD-3: test of gross motor development, 3rd edition
